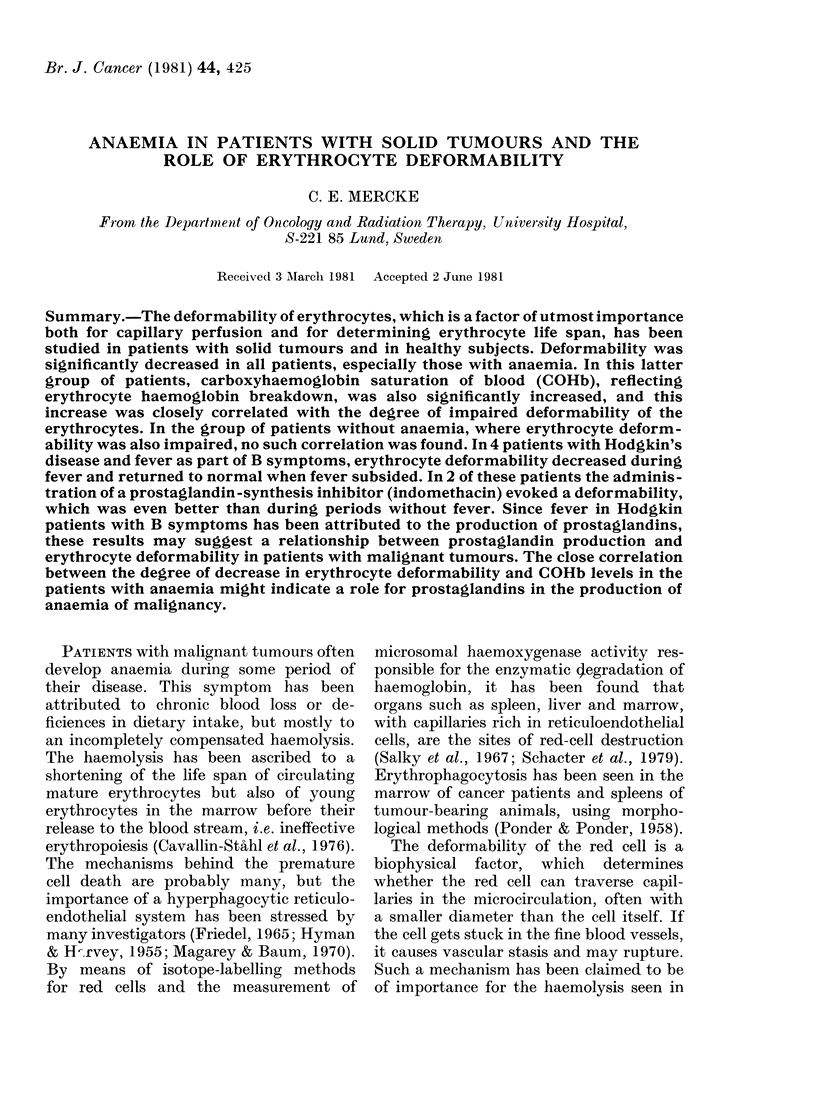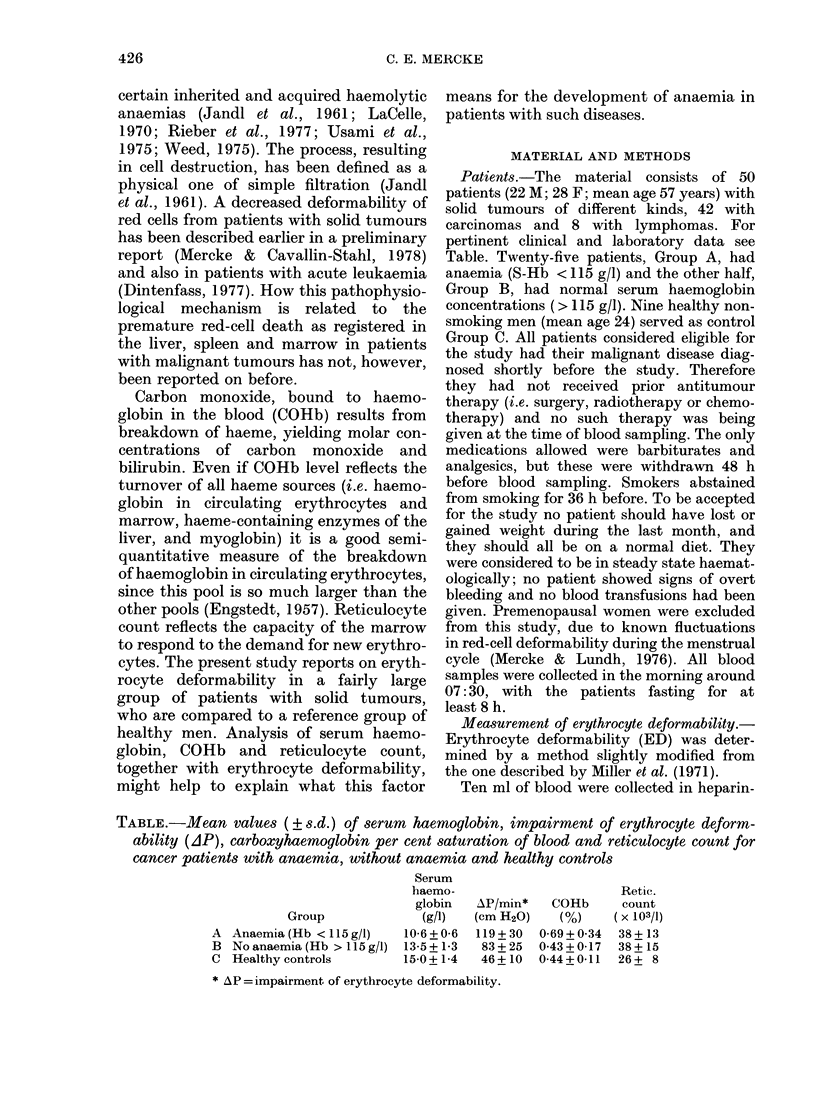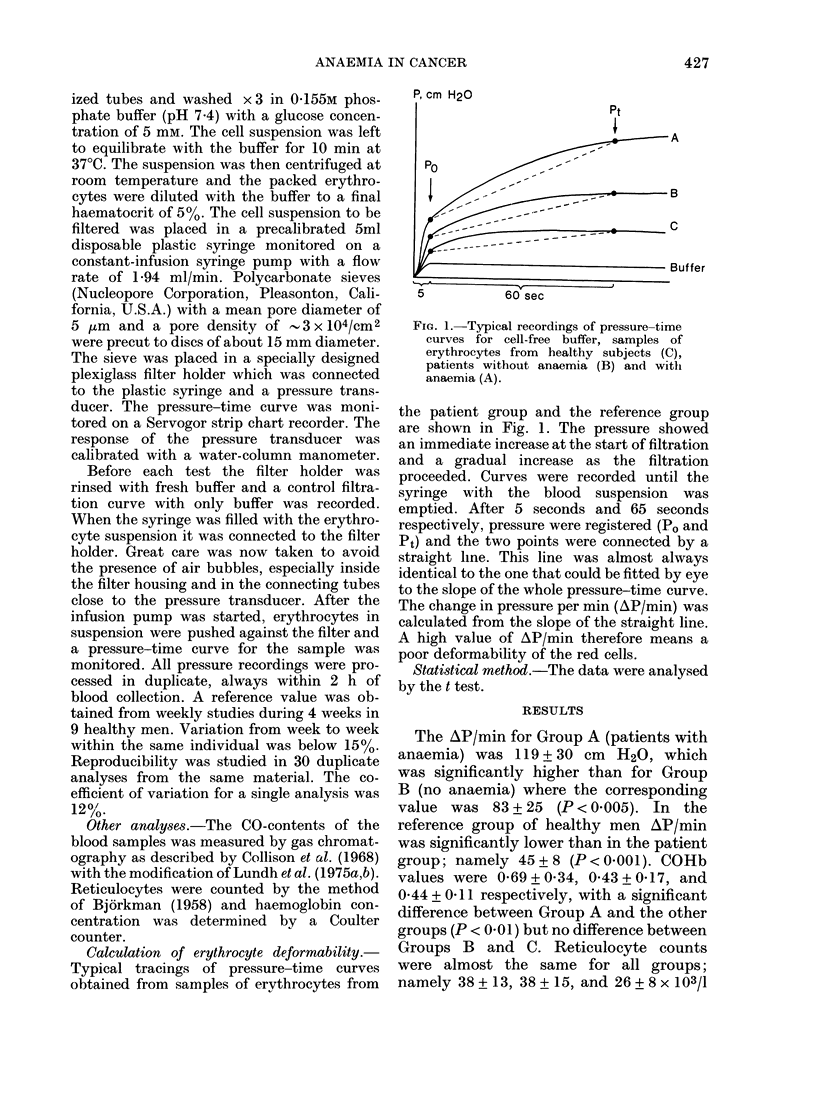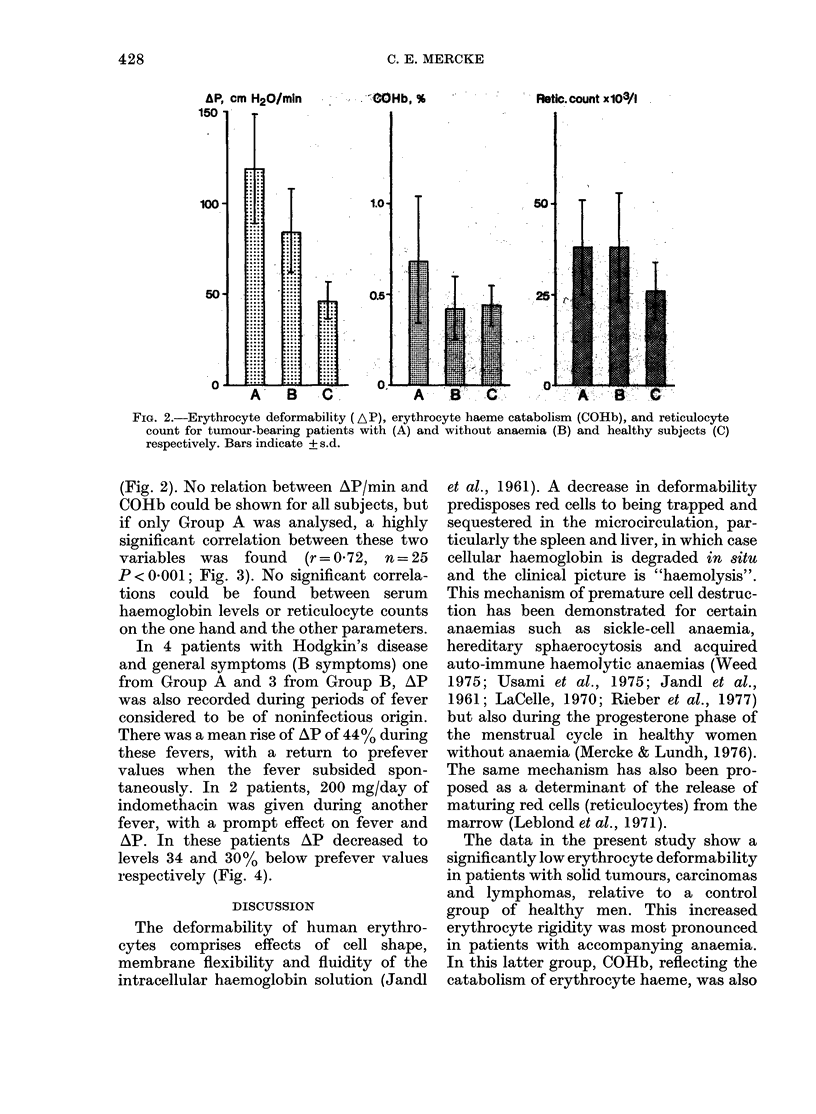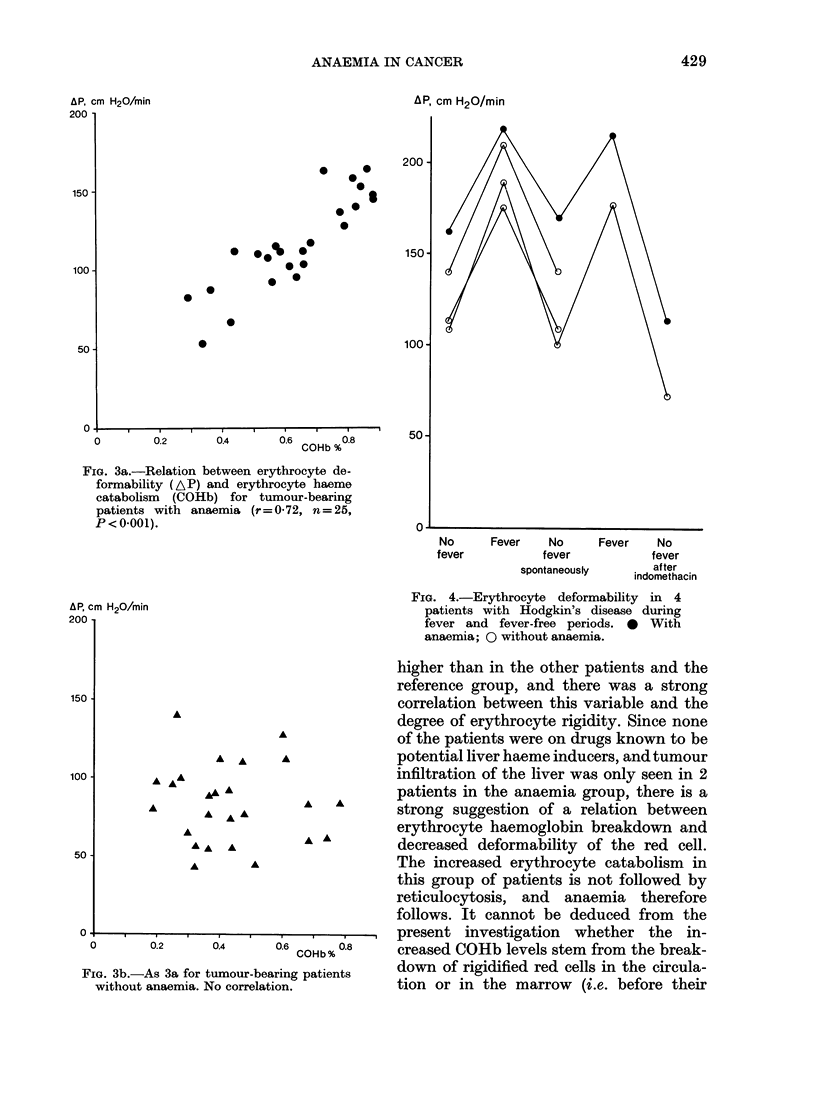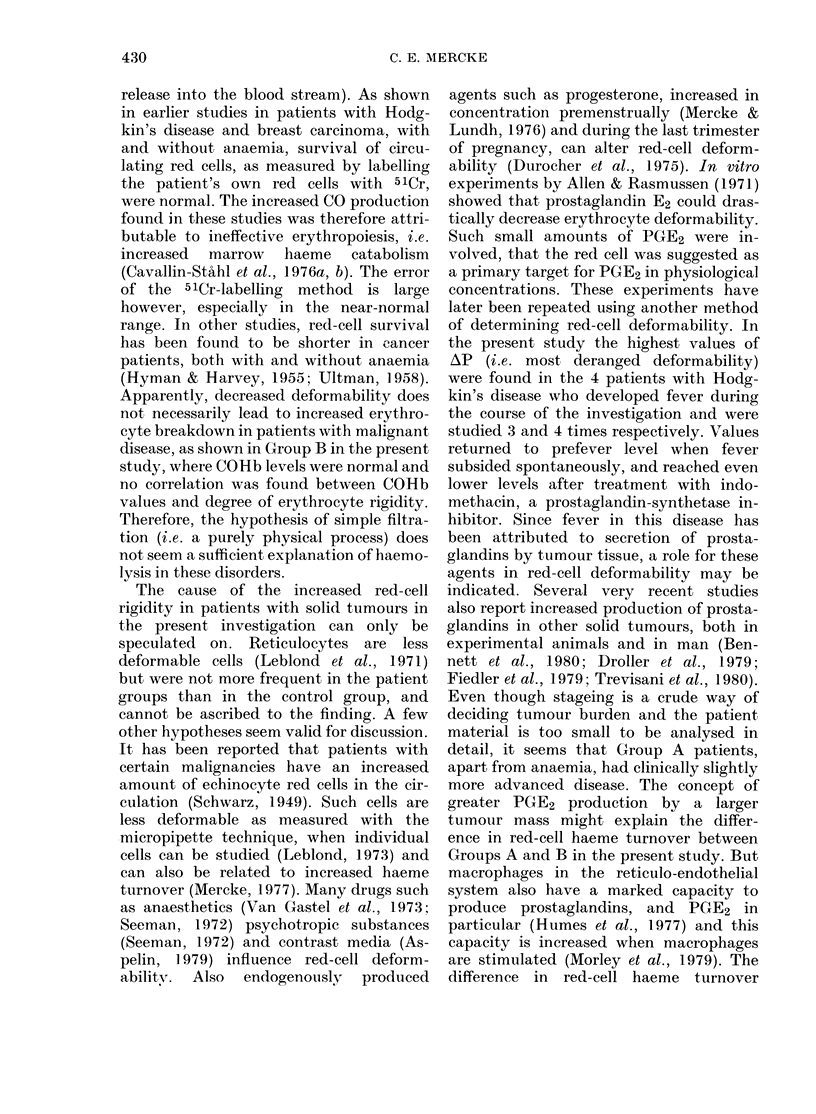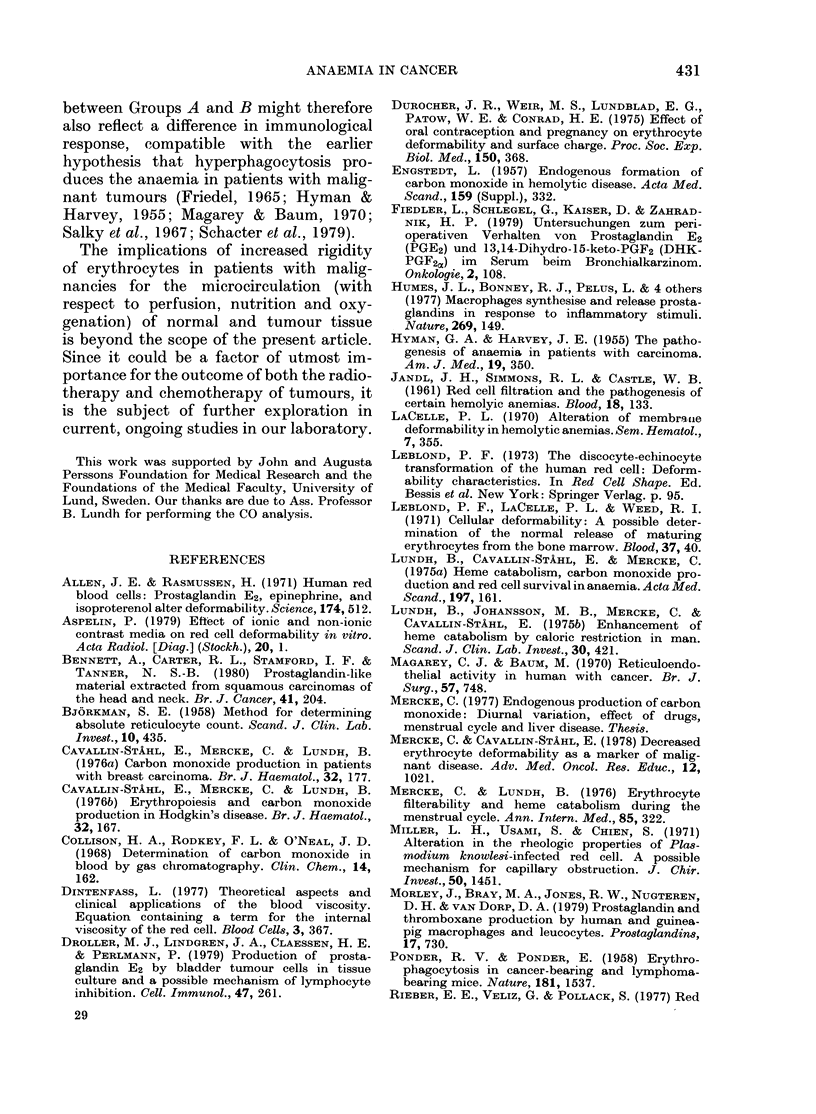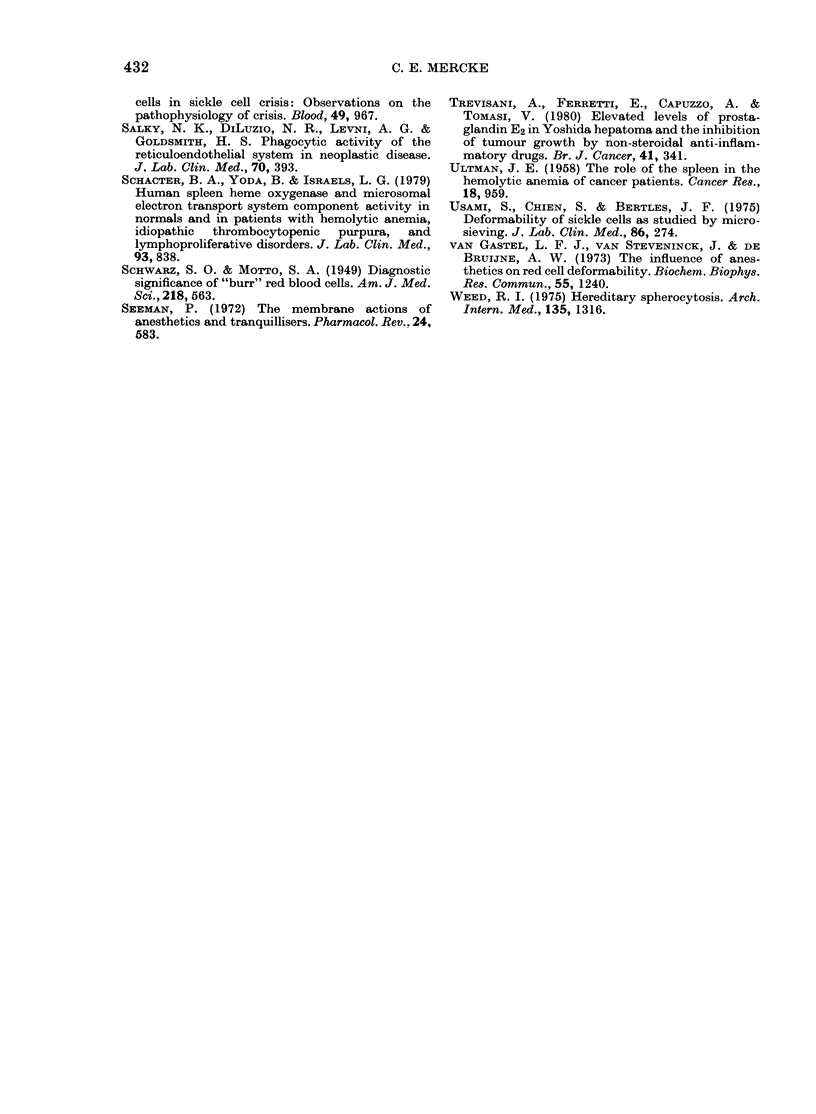# Anaemia in patients with solid tumours and the role of erythrocyte deformability.

**DOI:** 10.1038/bjc.1981.201

**Published:** 1981-09

**Authors:** C. E. Mercke

## Abstract

The deformability of erythrocytes, which is a factor of utmost importance both for capillary perfusion and for determining erythrocyte life span, has been studied in patients with solid tumours and in healthy subjects. Deformability was significantly decreased in all patients, especially those with anaemia. In this latter group of patients, carboxyhaemoglobin saturation of blood (COHb), reflecting erythrocyte haemoglobin breakdown, was also significantly increased, and this increase was closely correlated with the degree of impaired deformability of the erythrocytes. In the group of patients without anaemia, where erythrocyte deformability was also impaired, no such correlation was found. In 4 patients with Hodgkin's disease and fever as part of B symptoms, erythrocyte deformability decreased during fever and returned to normal when fever subsided. In 2 of these patients the administration of a prostaglandin-synthesis inhibitor (indomethacin) evoked a deformability, which was even better than during periods without fever. Since fever in Hodgkin patients with B symptoms has been attributed to the production of prostaglandins, these results may suggest a relationship between prostaglandin production and erythrocyte deformability in patients with malignant tumours. The close correlation between the degree of decrease in erythrocyte deformability and COHb levels in the patients with anaemia might indicate a role for prostaglandins in the production of anaemia of malignancy.


*Br. J. Cancer* (1981) **44**, 425

### ANAEMIA IN PATIENTS WITH SOLID TUMOURS AND THE ROLE OF ERYTHROCYTE DEFORMABILITY

C. E. MERCKE

*From the Department of Oncology and Radiation Therapy, University Hospital, S-221 85 Lund, Sweden*

Received 3 March 1981 Accepted 2 June 1981

**Summary.—The deformability of erythrocytes, which is a factor of utmost importance both for capillary perfusion and for determining erythrocyte life span, has been studied in patients with solid tumours and in healthy subjects. Deformability was significantly decreased in all patients, especially those with anaemia. In this latter group of patients, carboxyhaemoglobin saturation of blood (COHb), reflecting erythrocyte haemoglobin breakdown, was also significantly increased, and this increase was closely correlated with the degree of impaired deformability of the erythrocytes. In the group of patients without anaemia, where erythrocyte deformability was also impaired, no such correlation was found. In 4 patients with Hodgkin's disease and fever as part of B symptoms, erythrocyte deformability decreased during fever and returned to normal when fever subsided. In 2 of these patients the administration of a prostaglandin-synthesis inhibitor (indomethacin) evoked a deformability, which was even better than during periods without fever. Since fever in Hodgkin patients with B symptoms has been attributed to the production of prostaglandins, these results may suggest a relationship between prostaglandin production and erythrocyte deformability in patients with malignant tumours. The close correlation between the degree of decrease in erythrocyte deformability and COHb levels in the patients with anaemia might indicate a role for prostaglandins in the production of anaemia of malignancy.**

PATIENTS with malignant tumours often develop anaemia during some period of their disease. This symptom has been attributed to chronic blood loss or deficiences in dietary intake, but mostly to an incompletely compensated haemolysis. The haemolysis has been ascribed to a shortening of the life span of circulating mature erythrocytes but also of young erythrocytes in the marrow before their release to the blood stream, *i.e.* ineffective erythropoiesis (Cavallin-Ståhl *et al.*, 1976). The mechanisms behind the premature cell death are probably many, but the importance of a hyperphagocytic reticulo-endothelial system has been stressed by many investigators (Friedel, 1965; Hyman & Harvey, 1955; Magarey & Baum, 1970). By means of isotope-labelling methods for red cells and the measurement of microsomal haemoxygenase activity responsible for the enzymatic degradation of haemoglobin, it has been found that organs such as spleen, liver and marrow, with capillaries rich in reticuloendothelial cells, are the sites of red-cell destruction (Salky *et al.*, 1967; Schacter *et al.*, 1979). Erythrophagocytosis has been seen in the marrow of cancer patients and spleens of tumour-bearing animals, using morphological methods (Ponder & Ponder, 1958).

The deformability of the red cell is a biophysical factor, which determines whether the red cell can traverse capillaries in the microcirculation, often with a smaller diameter than the cell itself. If the cell gets stuck in the fine blood vessels, it causes vascular stasis and may rupture. Such a mechanism has been claimed to be of importance for the haemolysis seen in certain inherited and acquired haemolytic anaemias (Jandl *et al.*, 1961; LaCelle, 1970; Rieber *et al.*, 1977; Usami *et al.*, 1975; Weed, 1975). The process, resulting in cell destruction, has been defined as a physical one of simple filtration (Jandl *et al.*, 1961). A decreased deformability of red cells from patients with solid tumours has been described earlier in a preliminary report (Mercke & Cavallin-Stahl, 1978) and also in patients with acute leukaemia (Dintenfass, 1977). How this pathophysiological mechanism is related to the premature red-cell death as registered in the liver, spleen and marrow in patients with malignant tumours has not, however, been reported on before.

Carbon monoxide, bound to haemoglobin in the blood (COHb) results from breakdown of haeme, yielding molar concentrations of carbon monoxide and bilirubin. Even if COHb level reflects the turnover of all haeme sources (*i.e.* haemoglobin in circulating erythrocytes and marrow, haeme-containing enzymes of the liver, and myoglobin) it is a good semi-quantitative measure of the breakdown of haemoglobin in circulating erythrocytes, since this pool is so much larger than the other pools (Engstedt, 1957). Reticulocyte count reflects the capacity of the marrow to respond to the demand for new erythrocytes. The present study reports on erythrocyte deformability in a fairly large group of patients with solid tumours, who are compared to a reference group of healthy men. Analysis of serum haemoglobin, COHb and reticulocyte count, together with erythrocyte deformability, might help to explain what this factor means for the development of anaemia in patients with such diseases.

### MATERIAL AND METHODS

*Patients.*—The material consists of 50 patients (22 M; 28 F; mean age 57 years) with solid tumours of different kinds, 42 with carcinomas and 8 with lymphomas. For pertinent clinical and laboratory data see Table. Twenty-five patients, Group A, had anaemia (S-Hb < 115 g/l) and the other half, Group B, had normal serum haemoglobin concentrations ( > 115 g/l). Nine healthy non-smoking men (mean age 24) served as control Group C. All patients considered eligible for the study had their malignant disease diagnosed shortly before the study. Therefore they had not received prior antitumour therapy (*i.e.* surgery, radiotherapy or chemotherapy) and no such therapy was being given at the time of blood sampling. The only medications allowed were barbiturates and analgesics, but these were withdrawn 48 h before blood sampling. Smokers abstained from smoking for 36 h before. To be accepted for the study no patient should have lost or gained weight during the last month, and they should all be on a normal diet. They were considered to be in steady state haematologically; no patient showed signs of overt bleeding and no blood transfusions had been given. Premenopausal women were excluded from this study, due to known fluctuations in red-cell deformability during the menstrual cycle (Mercke & Lundh, 1976). All blood samples were collected in the morning around 07:30, with the patients fasting for at least 8 h.

*Measurement of erythrocyte deformability.*—Erythrocyte deformability (ED) was determined by a method slightly modified from the one described by Miller *et al.* (1971).

Ten ml of blood were collected in heparin-

TABLE.—*Mean values* ($\pm$ *s.d.*) *of serum haemoglobin, impairment of erythrocyte deformability* ($\Delta P$), *carboxyhaemoglobin per cent saturation of blood and reticulocyte count for cancer patients with anaemia, without anaemia and healthy controls*

| Group | | Serum haemoglobin (g/l) | ΔP/min* (cm $H_2O$) | COHb (%) | Retic. count ($\times 10^3$/l) |
|---|---|---|---|---|---|
| A | Anaemia (Hb < 115 g/l) | 10·6 ± 0·6 | 119 ± 30 | 0·69 ± 0·34 | 38 ± 13 |
| B | No anaemia (Hb > 115 g/l) | 13·5 ± 1·3 | 83 ± 25 | 0·43 ± 0·17 | 38 ± 15 |
| C | Healthy controls | 15·0 ± 1·4 | 46 ± 10 | 0·44 ± 0·11 | 26 ± 8 |

* ΔP = impairment of erythrocyte deformability.

ized tubes and washed ×3 in 0·155M phosphate buffer (pH 7·4) with a glucose concentration of 5 mM. The cell suspension was left to equilibrate with the buffer for 10 min at 37°C. The suspension was then centrifuged at room temperature and the packed erythrocytes were diluted with the buffer to a final haematocrit of 5%. The cell suspension to be filtered was placed in a precalibrated 5ml disposable plastic syringe monitored on a constant-infusion syringe pump with a flow rate of 1·94 ml/min. Polycarbonate sieves (Nucleopore Corporation, Pleasonton, California, U.S.A.) with a mean pore diameter of 5 $\mu$m and a pore density of $\sim 3 \times 10^4/cm^2$ were precut to discs of about 15 mm diameter. The sieve was placed in a specially designed plexiglass filter holder which was connected to the plastic syringe and a pressure transducer. The pressure–time curve was monitored on a Servogor strip chart recorder. The response of the pressure transducer was calibrated with a water-column manometer.

Before each test the filter holder was rinsed with fresh buffer and a control filtration curve with only buffer was recorded. When the syringe was filled with the erythrocyte suspension it was connected to the filter holder. Great care was now taken to avoid the presence of air bubbles, especially inside the filter housing and in the connecting tubes close to the pressure transducer. After the infusion pump was started, erythrocytes in suspension were pushed against the filter and a pressure–time curve for the sample was monitored. All pressure recordings were processed in duplicate, always within 2 h of blood collection. A reference value was obtained from weekly studies during 4 weeks in 9 healthy men. Variation from week to week within the same individual was below 15%. Reproducibility was studied in 30 duplicate analyses from the same material. The coefficient of variation for a single analysis was 12%.

*Other analyses.*—The CO-contents of the blood samples was measured by gas chromatography as described by Collison *et al.* (1968) with the modification of Lundh *et al.* (1975*a*,*b*). Reticulocytes were counted by the method of Björkman (1958) and haemoglobin concentration was determined by a Coulter counter.

*Calculation of erythrocyte deformability.*—Typical tracings of pressure–time curves obtained from samples of erythrocytes from

FIG. 1.—Typical recordings of pressure–time curves for cell-free buffer, samples of erythrocytes from healthy subjects (C), patients without anaemia (B) and with anaemia (A).

the patient group and the reference group are shown in Fig. 1. The pressure showed an immediate increase at the start of filtration and a gradual increase as the filtration proceeded. Curves were recorded until the syringe with the blood suspension was emptied. After 5 seconds and 65 seconds respectively, pressure were registered ($P_0$ and $P_t$) and the two points were connected by a straight line. This line was almost always identical to the one that could be fitted by eye to the slope of the whole pressure–time curve. The change in pressure per min ($\Delta$P/min) was calculated from the slope of the straight line. A high value of $\Delta$P/min therefore means a poor deformability of the red cells.

*Statistical method.*—The data were analysed by the *t* test.

### RESULTS

The $\Delta$P/min for Group A (patients with anaemia) was $119 \pm 30$ cm $H_2O$, which was significantly higher than for Group B (no anaemia) where the corresponding value was $83 \pm 25$ ($P < 0{\cdot}005$). In the reference group of healthy men $\Delta$P/min was significantly lower than in the patient group; namely $45 \pm 8$ ($P < 0{\cdot}001$). COHb values were $0{\cdot}69 \pm 0{\cdot}34$, $0{\cdot}43 \pm 0{\cdot}17$, and $0{\cdot}44 \pm 0{\cdot}11$ respectively, with a significant difference between Group A and the other groups ($P < 0{\cdot}01$) but no difference between Groups B and C. Reticulocyte counts were almost the same for all groups; namely $38 \pm 13$, $38 \pm 15$, and $26 \pm 8 \times 10^3$/l

FIG. 2.—Erythrocyte deformability ($\triangle$P), erythrocyte haeme catabolism (COHb), and reticulocyte count for tumour-bearing patients with (A) and without anaemia (B) and healthy subjects (C) respectively. Bars indicate $\pm$s.d.

(Fig. 2). No relation between ΔP/min and COHb could be shown for all subjects, but if only Group A was analysed, a highly significant correlation between these two variables was found ($r = 0{\cdot}72$, $n = 25$ $P < 0{\cdot}001$; Fig. 3). No significant correlations could be found between serum haemoglobin levels or reticulocyte counts on the one hand and the other parameters.

In 4 patients with Hodgkin's disease and general symptoms (B symptoms) one from Group A and 3 from Group B, ΔP was also recorded during periods of fever considered to be of noninfectious origin. There was a mean rise of ΔP of 44% during these fevers, with a return to prefever values when the fever subsided spontaneously. In 2 patients, 200 mg/day of indomethacin was given during another fever, with a prompt effect on fever and ΔP. In these patients ΔP decreased to levels 34 and 30% below prefever values respectively (Fig. 4).

### DISCUSSION

The deformability of human erythrocytes comprises effects of cell shape, membrane flexibility and fluidity of the intracellular haemoglobin solution (Jandl *et al.*, 1961). A decrease in deformability predisposes red cells to being trapped and sequestered in the microcirculation, particularly the spleen and liver, in which case cellular haemoglobin is degraded *in situ* and the clinical picture is "haemolysis". This mechanism of premature cell destruction has been demonstrated for certain anaemias such as sickle-cell anaemia, hereditary sphaerocytosis and acquired auto-immune haemolytic anaemias (Weed 1975; Usami *et al.*, 1975; Jandl *et al.*, 1961; LaCelle, 1970; Rieber *et al.*, 1977) but also during the progesterone phase of the menstrual cycle in healthy women without anaemia (Mercke & Lundh, 1976). The same mechanism has also been proposed as a determinant of the release of maturing red cells (reticulocytes) from the marrow (Leblond *et al.*, 1971).

The data in the present study show a significantly low erythrocyte deformability in patients with solid tumours, carcinomas and lymphomas, relative to a control group of healthy men. This increased erythrocyte rigidity was most pronounced in patients with accompanying anaemia. In this latter group, COHb, reflecting the catabolism of erythrocyte haeme, was also

FIG. 3a.—Relation between erythrocyte deformability (ΔP) and erythrocyte haeme catabolism (COHb) for tumour-bearing patients with anaemia ($r = 0{\cdot}72$, $n = 25$, $P < 0{\cdot}001$).

FIG. 3b.—As 3a for tumour-bearing patients without anaemia. No correlation.

FIG. 4.—Erythrocyte deformability in 4 patients with Hodgkin's disease during fever and fever-free periods. ● With anaemia; ○ without anaemia.

higher than in the other patients and the reference group, and there was a strong correlation between this variable and the degree of erythrocyte rigidity. Since none of the patients were on drugs known to be potential liver haeme inducers, and tumour infiltration of the liver was only seen in 2 patients in the anaemia group, there is a strong suggestion of a relation between erythrocyte haemoglobin breakdown and decreased deformability of the red cell. The increased erythrocyte catabolism in this group of patients is not followed by reticulocytosis, and anaemia therefore follows. It cannot be deduced from the present investigation whether the increased COHb levels stem from the breakdown of rigidified red cells in the circulation or in the marrow (*i.e.* before their release into the blood stream). As shown in earlier studies in patients with Hodgkin's disease and breast carcinoma, with and without anaemia, survival of circulating red cells, as measured by labelling the patient's own red cells with $^{51}Cr$, were normal. The increased CO production found in these studies was therefore attributable to ineffective erythropoiesis, *i.e.* increased marrow haeme catabolism (Cavallin-Ståhl *et al.*, 1976*a*, *b*). The error of the $^{51}Cr$-labelling method is large however, especially in the near-normal range. In other studies, red-cell survival has been found to be shorter in cancer patients, both with and without anaemia (Hyman & Harvey, 1955; Ultman, 1958). Apparently, decreased deformability does not necessarily lead to increased erythrocyte breakdown in patients with malignant disease, as shown in Group B in the present study, where COHb levels were normal and no correlation was found between COHb values and degree of erythrocyte rigidity. Therefore, the hypothesis of simple filtration (*i.e.* a purely physical process) does not seem a sufficient explanation of haemolysis in these disorders.

The cause of the increased red-cell rigidity in patients with solid tumours in the present investigation can only be speculated on. Reticulocytes are less deformable cells (Leblond *et al.*, 1971) but were not more frequent in the patient groups than in the control group, and cannot be ascribed to the finding. A few other hypotheses seem valid for discussion. It has been reported that patients with certain malignancies have an increased amount of echinocyte red cells in the circulation (Schwarz, 1949). Such cells are less deformable as measured with the micropipette technique, when individual cells can be studied (Leblond, 1973) and can also be related to increased haeme turnover (Mercke, 1977). Many drugs such as anaesthetics (Van Gastel *et al.*, 1973; Seeman, 1972) psychotropic substances (Seeman, 1972) and contrast media (Aspelin, 1979) influence red-cell deformability. Also endogenously produced agents such as progesterone, increased in concentration premenstrually (Mercke & Lundh, 1976) and during the last trimester of pregnancy, can alter red-cell deformability (Durocher *et al.*, 1975). *In vitro* experiments by Allen & Rasmussen (1971) showed that prostaglandin $E_2$ could drastically decrease erythrocyte deformability. Such small amounts of $PGE_2$ were involved, that the red cell was suggested as a primary target for $PGE_2$ in physiological concentrations. These experiments have later been repeated using another method of determining red-cell deformability. In the present study the highest values of $\Delta P$ (*i.e.* most deranged deformability) were found in the 4 patients with Hodgkin's disease who developed fever during the course of the investigation and were studied 3 and 4 times respectively. Values returned to prefever level when fever subsided spontaneously, and reached even lower levels after treatment with indomethacin, a prostaglandin-synthetase inhibitor. Since fever in this disease has been attributed to secretion of prostaglandins by tumour tissue, a role for these agents in red-cell deformability may be indicated. Several very recent studies also report increased production of prostaglandins in other solid tumours, both in experimental animals and in man (Bennett *et al.*, 1980; Droller *et al.*, 1979; Fiedler *et al.*, 1979; Trevisani *et al.*, 1980). Even though stageing is a crude way of deciding tumour burden and the patient material is too small to be analysed in detail, it seems that Group A patients, apart from anaemia, had clinically slightly more advanced disease. The concept of greater $PGE_2$ production by a larger tumour mass might explain the difference in red-cell haeme turnover between Groups A and B in the present study. But macrophages in the reticulo-endothelial system also have a marked capacity to produce prostaglandins, and $PGE_2$ in particular (Humes *et al.*, 1977) and this capacity is increased when macrophages are stimulated (Morley *et al.*, 1979). The difference in red-cell haeme turnover between Groups $A$ and $B$ might therefore also reflect a difference in immunological response, compatible with the earlier hypothesis that hyperphagocytosis produces the anaemia in patients with malignant tumours (Friedel, 1965; Hyman & Harvey, 1955; Magarey & Baum, 1970; Salky *et al.*, 1967; Schacter *et al.*, 1979).

The implications of increased rigidity of erythrocytes in patients with malignancies for the microcirculation (with respect to perfusion, nutrition and oxygenation) of normal and tumour tissue is beyond the scope of the present article. Since it could be a factor of utmost importance for the outcome of both the radiotherapy and chemotherapy of tumours, it is the subject of further exploration in current, ongoing studies in our laboratory.

This work was supported by John and Augusta Perssons Foundation for Medical Research and the Foundations of the Medical Faculty, University of Lund, Sweden. Our thanks are due to Ass. Professor B. Lundh for performing the CO analysis.

### REFERENCES

ALLEN, J. E. & RASMUSSEN, H. (1971) Human red blood cells: Prostaglandin $E_2$, epinephrine, and isoproterenol alter deformability. *Science*, **174**, 512.

ASPELIN, P. (1979) Effect of ionic and non-ionic contrast media on red cell deformability *in vitro*. *Acta Radiol. [Diag.] (Stockh.)*, **20**, 1.

BENNETT, A., CARTER, R. L., STAMFORD, I. F. & TANNER, N. S.-B. (1980) Prostaglandin-like material extracted from squamous carcinomas of the head and neck. *Br. J. Cancer*, **41**, 204.

BJÖRKMAN, S. E. (1958) Method for determining absolute reticulocyte count. *Scand. J. Clin. Lab. Invest.*, **10**, 435.

CAVALLIN-STÅHL, E., MERCKE, C. & LUNDH, B. (1976*a*) Carbon monoxide production in patients with breast carcinoma. *Br. J. Haematol.*, **32**, 177.

CAVALLIN-STÅHL, E., MERCKE, C. & LUNDH, B. (1976*b*) Erythropoiesis and carbon monoxide production in Hodgkin's disease. *Br. J. Haematol.*, **32**, 167.

COLLISON, H. A., RODKEY, F. L. & O'NEAL, J. D. (1968) Determination of carbon monoxide in blood by gas chromatography. *Clin. Chem.*, **14**, 162.

DINTENFASS, L. (1977) Theoretical aspects and clinical applications of the blood viscosity. Equation containing a term for the internal viscosity of the red cell. *Blood Cells*, **3**, 367.

DROLLER, M. J., LINDGREN, J. A., CLAESSEN, H. E. & PERLMANN, P. (1979) Production of prostaglandin $E_2$ by bladder tumour cells in tissue culture and a possible mechanism of lymphocyte inhibition. *Cell. Immunol.*, **47**, 261.

DUROCHER, J. R., WEIR, M. S., LUNDBLAD, E. G., PATOW, W. E. & CONRAD, H. E. (1975) Effect of oral contraception and pregnancy on erythrocyte deformability and surface charge. *Proc. Soc. Exp. Biol. Med.*, **150**, 368.

ENGSTEDT, L. (1957) Endogenous formation of carbon monoxide in hemolytic disease. *Acta Med. Scand.*, **159** (Suppl.), 332.

FIEDLER, L., SCHLEGEL, G., KAISER, D. & ZAHRADNIK, H. P. (1979) Untersuchungen zum perioperativen Verhalten von Prostaglandin $E_2$ ($PGE_2$) und 13,14-Dihydro-15-keto-$PGF_2$ (DHK-$PGF_{2\alpha}$) im Serum beim Bronchialkarzinom. *Onkologie*, **2**, 108.

HUMES, J. L., BONNEY, R. J., PELUS, L. & 4 others (1977) Macrophages synthesise and release prostaglandins in response to inflammatory stimuli. *Nature*, **269**, 149.

HYMAN, G. A. & HARVEY, J. E. (1955) The pathogenesis of anaemia in patients with carcinoma. *Am. J. Med.*, **19**, 350.

JANDL, J. H., SIMMONS, R. L. & CASTLE, W. B. (1961) Red cell filtration and the pathogenesis of certain hemolyic anemias. *Blood*, **18**, 133.

LACELLE, P. L. (1970) Alteration of membrane deformability in hemolytic anemias. *Sem. Hematol.*, **7**, 355.

LEBLOND, P. F. (1973) The discocyte-echinocyte transformation of the human red cell: Deformability characteristics. In *Red Cell Shape*. Ed. Bessis *et al.* New York: Springer Verlag. p. 95.

LEBLOND, P. F., LACELLE, P. L. & WEED, R. I. (1971) Cellular deformability: A possible determination of the normal release of maturing erythrocytes from the bone marrow. *Blood*, **37**, 40.

LUNDH, B., CAVALLIN-STÅHL, E. & MERCKE, C. (1975*a*) Heme catabolism, carbon monoxide production and red cell survival in anaemia. *Acta Med. Scand.*, **197**, 161.

LUNDH, B., JOHANSSON, M. B., MERCKE, C. & CAVALLIN-STÅHL, E. (1975*b*) Enhancement of heme catabolism by caloric restriction in man. *Scand. J. Clin. Lab. Invest.*, **30**, 421.

MAGAREY, C. J. & BAUM, M. (1970) Reticuloendothelial activity in human with cancer. *Br. J. Surg.*, **57**, 748.

MERCKE, C. (1977) Endogenous production of carbon monoxide: Diurnal variation, effect of drugs, menstrual cycle and liver disease. *Thesis*.

MERCKE, C. & CAVALLIN-STÅHL, E. (1978) Decreased erythrocyte deformability as a marker of malignant disease. *Adv. Med. Oncol. Res. Educ.*, **12**, 1021.

MERCKE, C. & LUNDH, B. (1976) Erythrocyte filterability and heme catabolism during the menstrual cycle. *Ann. Intern. Med.*, **85**, 322.

MILLER, L. H., USAMI, S. & CHIEN, S. (1971) Alteration in the rheologic properties of *Plasmodium knowlesi*-infected red cell. A possible mechanism for capillary obstruction. *J. Chir. Invest.*, **50**, 1451.

MORLEY, J., BRAY, M. A., JONES, R. W., NUGTEREN, D. H. & VAN DORP, D. A. (1979) Prostaglandin and thromboxane production by human and guineapig macrophages and leucocytes. *Prostaglandins*, **17**, 730.

PONDER, R. V. & PONDER, E. (1958) Erythrophagocytosis in cancer-bearing and lymphomabearing mice. *Nature*, **181**, 1537.

RIEBER, E. E., VELIZ, G. & POLLACK, S. (1977) Red cells in sickle cell crisis: Observations on the pathophysiology of crisis. *Blood*, **49**, 967.

SALKY, N. K., DILUZIO, N. R., LEVNI, A. G. & GOLDSMITH, H. S. Phagocytic activity of the reticuloendothelial system in neoplastic disease. *J. Lab. Clin. Med.*, **70**, 393.

SCHACTER, B. A., YODA, B. & ISRAELS, L. G. (1979) Human spleen heme oxygenase and microsomal electron transport system component activity in normals and in patients with hemolytic anemia, idiopathic thrombocytopenic purpura, and lymphoproliferative disorders. *J. Lab. Clin. Med.*, **93**, 838.

SCHWARZ, S. O. & MOTTO, S. A. (1949) Diagnostic significance of "burr" red blood cells. *Am. J. Med. Sci.*, **218**, 563.

SEEMAN, P. (1972) The membrane actions of anesthetics and tranquillisers. *Pharmacol. Rev.*, **24**, 583.

TREVISANI, A., FERRETTI, E., CAPUZZO, A. & TOMASI, V. (1980) Elevated levels of prostaglandin $E_2$ in Yoshida hepatoma and the inhibition of tumour growth by non-steroidal anti-inflammatory drugs. *Br. J. Cancer*, **41**, 341.

ULTMAN, J. E. (1958) The role of the spleen in the hemolytic anemia of cancer patients. *Cancer Res.*, **18**, 959.

USAMI, S., CHIEN, S. & BERTLES, J. F. (1975) Deformability of sickle cells as studied by microsieving. *J. Lab. Clin. Med.*, **86**, 274.

VAN GASTEL, L. F. J., VAN STEVENINCK, J. & DE BRUIJNE, A. W. (1973) The influence of anesthetics on red cell deformability. *Biochem. Biophys. Res. Commun.*, **55**, 1240.

WEED, R. I. (1975) Hereditary spherocytosis. *Arch. Intern. Med.*, **135**, 1316.